# A reassessment of global antenatal care coverage for improving maternal health using sub-Saharan Africa as a case study

**DOI:** 10.1371/journal.pone.0204822

**Published:** 2018-10-05

**Authors:** John Ele-Ojo Ataguba

**Affiliations:** Health Economics Unit, School of Public Health and Family Medicine, Health Sciences Faculty, University of Cape Town, Cape Town, South Africa; The University of Warwick, UNITED KINGDOM

## Abstract

**Background:**

Antenatal period is an opportunity for reaching pregnant women with vital interventions. In fact, antenatal care (ANC) coverage was an indicator for assessing progress towards the Millennium Development Goals. This paper applies a novel index of service coverage using ANC, which accounts for every ANC visit. An index of service coverage gap is also proposed. These indices are additively decomposable by population groups and they are sensitive to the receipt of more ANC visits below a defined threshold. These indices have also been generalised to account for the quality of services.

**Methods:**

Data from recent rounds of the Demographic and Health Survey (DHS) are used to reassess ANC service coverage in 35 sub-Saharan African countries. An index of ANC coverage was estimated. These countries were ranked, and their ranks are compared with those based on attaining at least four ANC visits (ANC4+).

**Findings:**

The index of ANC coverage reflected the level of service coverage in countries. Further, disparities exist in country ranking as some countries, e.g. Cameroon, Benin Republic and Nigeria are ranked better using the ANC4+ indicator but poorly using the proposed index. Also, Rwanda and Malawi are ranked better using the proposed index.

**Conclusion:**

The proposed ANC index allows for the assessment of progressive realisation, rooted in the move towards universal health coverage. In fact, the index reflects progress that countries make in increasing service coverage. This is because every ANC visit counts. Beyond ANC coverage, the proposed index is applicable to assessing service coverage generally including quality education.

## Introduction

There is a growing concern globally, especially within developing countries, to improve maternal and child health outcome indicators [1, 2]. The antenatal period provides an opportunity for reaching pregnant women with interventions that may be vital to their health and wellbeing and that of their children. In fact, evidence shows that “women from high-, medium- and low-resource settings valued having a positive pregnancy experience” ([3] p.86). While such a positive pregnancy experience is multidimensional, within the health system, women have expressed concern for flexible appointment systems and ensuring continuity in the provision of care with emphasis placed on ensuring privacy and providing quality time to build trust and a good relationship with health service providers. They also value having culturally sensitive, safe and effective health services [3, 4].

Antenatal care (ANC) provides an avenue for pregnant women to use services that contribute to a “positive pregnancy experience”. ANC coverage remains an important indicator of access and use of health care during pregnancy [1, 5–7]. In fact, it was used as an indicator for assessing maternal health in the context of the Millennium Development Goals (MDGs) [1]. Post MDGs, an unfinished agenda remains with the health of newborns and stillbirths [6]. Empirical studies have demonstrated the positive impact of antenatal care on child birthweight [8–11], early detection of foetal abnormalities including the diagnosis of growth retardation [7, 12] and reductions in maternal and neonatal morbidity and mortality [7, 13–15], for instance. However, there remains a debate about the adequacy or sufficiency of using ANC contacts alone without much reference to the quality of such contacts [16–18]. However, regular ANC contacts with qualified professionals still afford the opportunity to provide pregnant women with both preventative and treatment services such as treatment and management of hypertension and diabetes, distribution of insecticide-treated mosquito nets in malaria endemic places, prevention of mother-to-child transmission of HIV, etc. [1].

Prior to 2016, the World Health Organization (WHO) recommended a “reduced-visit” model of at least four ANC visits for pregnant women (in the case of uncomplicated pregnancies), with the first visit occurring in the first trimester (i.e. the first 12 weeks of conception) [3, 5]. Recently, however, a “standard” model of attaining at least eight ANC visits has been recommended (see Table 1). This is because evidence shows improvements in health outcomes and an increased likelihood of receiving effective maternal health interventions under the “standard” ANC model compared to the “reduced-visit” model [3].

**Table 1:** The ‘reduced’ and ‘standard’ World Health Organization’s ANC models.

‘Reduced’ ANC model	2016 ANC model (standard)
First trimester	
Visit 1: 8–12 weeks	Contact 1: up to 12 weeks
Second trimester	
Visit 2: 24–26 weeks	Contact 2: 20 weeks
	Contact 3: 26 weeks
Third trimester	
Visit 3: 32 weeks	Contact 4: 30 weeks
	Contact 5: 34 weeks
Visit 4: 36–38 weeks	Contact 6: 36 weeks
	Contact 7: 38 weeks
	Contact 8: 40 weeks
Return for delivery at 41 weeks if not given birth	

*Source*: World Health Organization (3)

Globally, the WHO reports ANC coverage (%) as the proportion of women aged 15–49 with a live birth in a given time period that attain at least one ANC visit and at least four visits (i.e. the ‘reduced’ ANC model) [19]. Inclusion of an indicator of having at least one ANC visit provides an opportunity to capture women with a live birth within a given period who were unable to attain more than one ANC visit. However, at least in a developing country context, as will be demonstrated later, these indicators, including an indicator for the “standard” ANC model, may not be sensitive to policies that have been able to increase the proportion of women that have attained between two and three ANC visits, for instance. This is because only the women that have attained the recommended minimum number of visits are counted. The proportion of pregnant women attaining at least four ANC visits (in the case of the reduced model) is also unable to adequately discriminate between countries with relatively similar proportions attaining at least four visits irrespective of the proportion attaining at least three ANC visits, for instance. For example, in Kenya and Uganda (2006–2013), while coverage with at least one ANC visit was nearly universal (>91%), coverage with at least four ANC visits was less than half (<49%) [20]. In these countries, any policy that aims at increasing the proportion of the women with at least one ANC visit will be close to the notion of “progressive realisation” within the Universal Health Coverage (UHC) discourse. In fact, the WHO notes that “large expansions in antenatal care coverage are still needed” especially in developing countries to achieve universalism [19] and this cannot be achieved at once but gradually.

On the assessment front, reporting at least four ANC visits remains the widely used global benchmark for antenatal care until recently when this was increased to eight ANC visits. While this relies directly on counting the number of visits, some authors have suggested alternative measures at the individual or aggregate population level that go beyond counting the contacts but reflecting the content of the ANC received. This includes the proportion of women attaining at least four ANC visits that report receiving the full set (or a defined set) of specific elements of care [16, 17]. In fact, a study has reported a significant association between the content and timing of care and preterm birth [18]. One of the major challenges with using indicators that account for the ‘content’ of the services or the depth of coverage is the absence of reliable routine data. However, the relatively well-established data on the number of ANC contacts could still be used to provide a much richer measure of ANC coverage that could be simple to compute and to interpret until the availability of a more comprehensive measure that takes the quality of ANC into account. This paper shall return to the challenging issue of assessing the quality of ANC services, like any other health service.

## Objectives

This paper builds on the traditional indicators of ANC coverage, particularly the ‘reduced’ ANC model (see Table 1) and proposes an index of ANC coverage that uses the number of ANC contacts over the “entire” distribution (0–4+ visits in this case. For noting, this can be extended easily to the “standard” ANC model of 0–8+ contacts). The proposed index is designed to be very simple to compute and to explain to policymakers and can be useful to compare countries and for monitoring progress in ANC coverage over time. The properties of this index are also presented in S1 Appendix. In the light of the debates around effective coverage [21], the paper also proposes an extension by developing a generalised index of ANC coverage that analysts can use to account for the quality of each ANC received and not just the number of ANC contacts.

## Conceptualising the reassessment of ANC coverage

Let *v*_*ij*_ represet the *i*th ANC visit for woman *j* aged 15–49 with a live birth within a given period such that:

$$v_{i}=\left\{ \begin{array}{l} 1\;if\;there\;was\;an\;ith\;visit\\ 0\;otherwise \end{array}\right.\;\mathrm{for}\;\mathrm{each}j$$ (1)

The total number of ANC visits (i.e. *n*_*j*_) for each woman aged 15–49 with a live birth within a given period is given as *V*_*j*_ = *n*_*j*_ = ∑_*i*_ *v*_*ij*_ such that *V*_*j*_ ≥ 0.

For now, let us ignore the quality of each ANC visit including the actual timing of each visit, as we shall return to this later. If we define *ρ*_*k*_ as the proportion of women aged 15–49 with a live birth within a given period with *k* ANC visits, and let *Y*_*k*_ be the cumulative proportion of women aged 15–49 with a live birth within a given period that attain at least *k* number of ANC visits, then,

$$Y_{k}=\left\{ \begin{array}{l} \sum_{i=k}^{n^{max}}\rho_{i}\\ 0 \end{array}\right.\;\begin{array}{l} ifn^{max}>k\\ Otherwise \end{array}$$ (2)

where *n*^max^ is the highest number of ANC visits attained by any woman aged 15–49 with a live birth within a given period.

If *m* is the recommended minimum number of ANC visits (e.g. four) for women aged 15–49 with a live birth within a given period, a measure (index) of ANC coverage is proposed as:

$$I_{ANC}=\frac{1}{m}\sum_{k=1}^{m}Y_{k}=\mu_{Y_{k}}$$ (3)

The corresponding ANC coverage gap index is written as:

$$G_{ANC}=1-I_{ANC}\equiv \frac{1}{m}\sum_{k=1}^{m}\left(1-Y_{k}\right)=1-\mu_{Y_{k}}$$ (4)

Theoretically, the value of each index will range between zero and one. When *I*_*ANC*_ → 0, it signifies that ANC coverage is extremely low irrespective of any minimum ANC visits (*m* > 0). While *I*_*ANC*_ → 1 signifies that for the specified recommended minimum number of ANC visits, ANC coverage is very high. Analogously, 0 ≤ *G*_*ANC*_ ≤ 1 such that *G*_*ANC*_ → 0 when there is an extremely high ANC coverage (i.e., minimal coverage gap) and *G*_*ANC*_ → 1 when the level of ANC coverage is extremely low.

Basically, when *I*_*ANC*_ → 1 (or *G*_*ANC*_ → 0) it means that 100% of women aged 15–49 with a live birth within a given period attained the recommended minimum number of ANC visits while a value of zero (or *G*_*ANC*_ → 1) implies that none of the women had any ANC visit (i.e., 100% of the women had no ANC visit). Essentially, countries should aim to minimise *G*_*ANC*_ (or maximise *I*_*ANC*_) for a *progressive* realisation of ANC coverage.

Based on the WHO’s ‘reduced’ ANC model of attaining at least four ANC visits for uncomplicated pregnancies, we can re-write Eq (3) as:

$$I_{ANC}=0.25\;\sum_{k=1}^{4}Y_{k}$$ (5)

Analogous expression can be written for the coverage gap index.

The index in Eq (5) is appealing because it is relatively easy to compute and to interpret. However, its values and the corresponding interpretation are not directly comparable to those of the proportion of pregnant women attaining at least four ANC visits. In fact, this index can be reported and complemented with the traditional indices of ANC service coverage (i.e. the coverage with at least one ANC visit and coverage with at least four or eight ANC visits). It provides policymakers with a complementary measure to use to assess the impact of policies over time, which may have had an impact but not on the number of visits or contacts above the recommended WHO minimum threshold(s).

**Fig 1:**
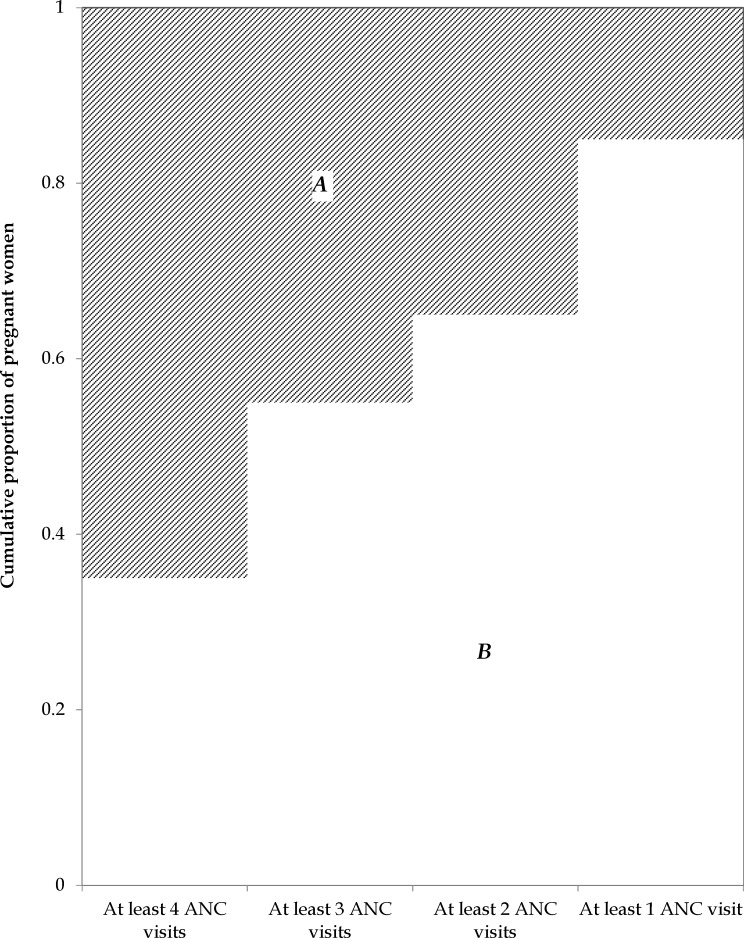
A measure of ANC coverage (*I*_*ANC*_) illustrated.

Pictorially, the ANC coverage index (*I*_*ANC*_) and coverage gap index (*G*_*ANC*_) can be computed as the area of the non-shaded and shaded portions in Fig 1, respectively. The vertical axis represents the cumulative proportion of women (aged 15–49) with a live birth that attain at least *k* number of ANC visits. The coverage index corresponds to:

$$I_{ANC}=\frac{B}{A+B}$$ (6)

while the corresponding ANC coverage gap index can be obtained as:

$$G_{ANC}=1-I_{ANC}=\frac{A}{A+B}$$ (7)

**Fig 2:**
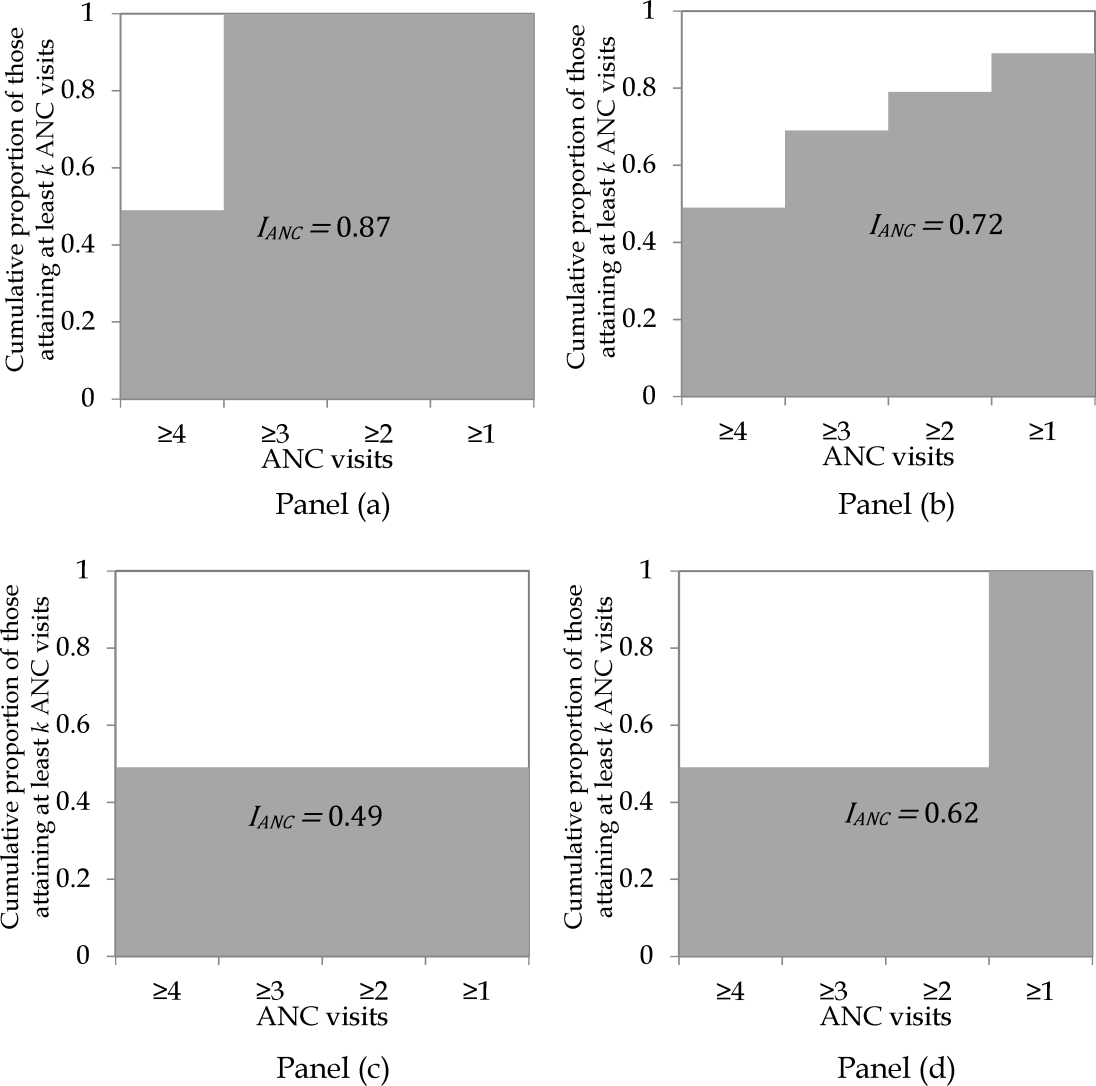
Illustrating different scenarios where 49.0% of women aged 15–49 with a live birth within a given period attained at least four ANC visits.

Fig 2 shows possible values of *I*_*ANC*_ for a case where 49% of women (aged 15–49) with a live birth within a given period had attained at least four ANC visits. Because *I*_*ANC*_ captures all visits, its values range between 0.49 (in panel c) and 0.87 (in panel a) in this case. So, it shows that ANC coverage in panel (a) is better than that in the rest of the panels. In fact, panel (c) has the worst level of ANC coverage. These differences are not accounted for when using at least four ANC visits as an indicator of ANC coverage.

Box 1 contains a summary of the steps involved in computing *I*_*ANC*_ and *G*_*ANC*_ from survey data, assuming a recommended minimum of four ANC visits.

**Box 1. Steps for computing the new index of ANC coverage (*I*_*ANC*_) and ANC coverage gap (*G*_*ANC*_)**

***Step 1***: Estimate the proportion of women aged 15–49 with a live birth within a given period that had only one ANC visit. Ideally, this should be to a formal health service provider
***Step 2***: Repeat step 1 to obtain the proportion of women that received two, three, and four or more ANC visits
***Step 3***: Estimate the *cumulative* proportion of women aged 15–49 with a live birth within a given period that received at least four ANC visits
***Step 4***: Repeat step 3 to obtain the *cumulative* proportion of women that received at least three, at least two, and at least one ANC visit.
***Step 5***: Add up all the *cumulative* proportions obtained in steps 3 and 4, then divide the sum by 4 (corresponding to the recommended minimum number of ANC visits) to obtain the proposed index of ANC coverage (i.e., *I*_*ANC*_)0
***Step 6***: ANC coverage gap is estimated as: *G*_*ANC*_ = 1 − *I*_*ANC*_.

The indices proposed in Box 1 can be computed using an alternative approach. Recall that *V*_*j*_ = *n*_*j*_ represents the total number of ANC visits for each woman aged 15–49 with a live birth within a given period.

We define a censored distribution of *V* as V* such that:

$$V_{j}^{*}=\left\{ \begin{array}{l} V_{j}\;if\;V_{j}<m\\ m\;if\;V_{j}\geq m \end{array}\right.$$ (8)

where *m* remains as defined previously.

The index, *I*_*ANC*_, can be estimated equivalently as:

$$I_{ANC}=\frac{1}{m*n}\sum_{j=1}^{n}V_{j}^{*}$$ (9)

where *n* is the total number of woman aged 15–49 with a live birth within a given period.

### Dominance testing (and ordering) for the proposed ANC coverage index

The proposed index (*I*_*ANC*_) could be used to make international comparison between countries (or generally, any comparison between two distributions). This could be of interest to policymakers and practitioners for designing appropriate interventions. To make unambiguous comparisons it is important to assess the statistical dominance of the distributions of ANC coverage. For example, statistical dominance test can be conducted for ANC coverage between the rural and the urban populations in a country, between two ethnic or racial groups or between two countries.

As an analogy, two distributions (*A* and *B*) may have the same index value but one might be preferred to the other (e.g. $I_{ANC}^{A}=I_{ANC}^{B}$ but $I_{ANC}^{A}≻I_{ANC}^{B}$). Also, $I_{ANC}^{A}>I_{ANC}^{B}$ yet distribution *B* may be considered as better than *A*. The test for dominance provides an avenue to make such assessments.

#### Necessary condition for statistical dominance

A necessary condition for distribution *A* (e.g., country or rural or urban location) to dominate distribution *B*, which is proposed in this paper, is that:

$$Y_{m-1}^{A}-Y_{m-1}^{B}\geq 0$$ (10)

where $Y_{m-1}^{A}$ is the cumulative proportion of women (in distribution *A*) aged 15–49 with live births within a given period that attain at least one visit less than the recommended minimum number of ANC visits. For the case of the WHO’s ‘reduced’ and ‘standard’ ANC model recommendations, this is equivalent to three and seven visits, respectively. In general, the subscript (*m* − 1) may be replaced with (*m* − *l*) where (0 ≤ *l* < *m*) represents the number adjudged by the analyst. In an extreme and strict case, *l* = 0 (see the case of the sufficient condition).

#### Sufficient condition for statistical dominance

Strictly speaking, distribution *B* is dominated by distribution *A* if

$$Y_{k}^{A}-Y_{k}^{B}\geq 0\;\forall k$$ (11)

where *Y*_*k*_ is the cumulative proportion of pregnant women attaining at least *k* ANC visits.

#### A generalised measure of ANC coverage: accounting for the quality of ANC services

The usual indicators of ANC coverage, including the attainment of at least four ANC visits, remain very informative and relevant in many contexts. Even the indicator of attaining at least one ANC visit is relevant in Africa [16]. However, such “[c]overage estimates… do not address the quality of that contact or whether it provided needed interventions…. Quality assessments of such services are an essential part of sound programme management” ([22] p.16). In fact, while higher ANC coverage is critical, the quality of care (including information on the services and interventions that are actually provided) and the timing of each visit also determine the extent to which such services are effective [7, 18, 22]. In many developing countries the quality of services provided remains low [23, 24], which often leads to poor maternal and child health outcomes. Thus, it is recommended that countries “identify gaps in coverage and quality of care” as a way to ensure that pregnant women receive appropriate needed care [22, 25], which extends *inter alia* beyond the number of contacts. However, there are challenges with the assessment of quality of ANC services, more so in developing countries where *inter alia* the data architecture is poorly developed [25]. Notwithstanding these issues, few studies have attempted to assess this within Africa (see for example, [16, 23, 26]) and beyond [8] using a selected set of interventions.

This paper recognises the debate surrounding the assessment of quality health services generally (and ANC services in particular), including its multidimensional nature. However, it does not delve into identifying how to measure and assess such as this is beyond the scope of the paper. It only provides a generalised framework, which can be used to assess ANC service coverage that takes the quality of such services for *each* ANC visit into account. For simplicity and ease of applicability, the measure of quality of ANC services assumed in this paper is scaled to range between zero and one, with one being the “best” quality.

If we define *q*_*ij*_ as a measure of the quality of ANC visit *i* for individual *j*, such that *q*_*ij*_ ∈ [0,1]. Briefly, *q*_*ij*_ = 0 is the worst possible quality of service or the absence of any service provided during an ANC visit. Also, *q*_*ij*_ = 1 is the best quality service that can be imagined conditional upon the available level of technology. The quality-adjusted *i*th ANC visit for individual *j* can then be written as:

$$v_{ij}q_{ij}$$ (12)

where *v*_*ij*_ remains as defined earlier and *v*_*ij*_*q*_*ij*_ ∈ [0,1].

In fact,

$$v_{ij}q_{ij}=\left\{ \begin{array}{l} q_{ij}\;if\;there\;was\;an\;ith\;visit\;(v_{ij}=1)\\ 0\;if\;v_{ij}=0\;or\;q_{ij}=0 \end{array}\right.$$ (13)

The “total” quality-adjusted ANC visits for each woman aged 15–49 with a live birth within a given period can be written as $V_{j}^{Adj}=\sum_{i}v_{ij}q_{ij}$. Thus, $V_{j}^{Adj}\leq V_{j}$.

Let the proportion of women aged 15–49 with a live birth within a given period with *k* quality-adjusted ANC visits be defined as:

$$\rho_{k}^{Adj}=\frac{1}{N}\sum_{j=1}^{N}v_{kj}q_{kj}$$ (14)

The cumulative proportion of women aged 15–49 with a live birth within a given period, adjusted for quality of services ($Y_{k}^{Adj}$), that attain at least *k* number of ANC visits can also be written as:

$$Y_{k}^{Adj}=\left\{ \begin{array}{l} \sum_{i=k}^{n^{max}}\rho_{i}^{Adj}\;\\ \;0 \end{array}\right.\begin{array}{l} ifn^{max}>k\\ Otherwise \end{array}$$ (15)

where *n*^max^ remains as defined.

The proposed measure of ANC coverage, generalised for quality of care, $I_{ANC}^{Adj}$, can be written as:

$$I_{ANC}^{Adj}=\frac{1}{m}\sum_{k=1}^{m}Y_{k}^{Adj}=\mu_{Y_{k}^{Adj}}$$ (16)

Subsequently, the quality-adjusted ANC coverage gap can be written as:

$$G_{ANC}^{Adj}=1-I_{ANC}^{Adj}=\frac{1}{m}\sum_{k=1}^{m}{(1-Y}_{k}^{Adj})=1-\mu_{Y_{k}^{Adj}}$$ (17)

For noting, the measure of ANC coverage in Eq (3) is equivalent to that in Eq (16) if we assume that *q*_*ij*_ = 1 ∀*i*,*j*. This means that *I*_*ANC*_ assumes that the quality of ANC services is perfect for all ANC visits. Eq (16) and (17) represent the generalised formulae for estimating ANC coverage and coverage gap, respectively. Because available data (e.g. the Demographic and Health Surveys) do not contain information on the quality of each ANC contact, this generalised index was applied indirectly in this paper by assuming perfect quality for all ANC contacts.

### Methodology

The proposed approach for reassessing ANC coverage is applied to data from Uganda, one of the African countries with a relatively high proportion of women that do not attain at least four ANC visits. The Uganda case study presents a detailed analysis of the coverage index (*I*_*ANC*_). In addition, the methodology is applied to data from 35 sub-Saharan African countries to assess the differences in ranking of countries compared to when the traditional indicator of attaining at least four ANC visits is used.

### Data

The Uganda Demographic and Health Survey 2016 (UDHS-2016) data are used for an in-depth assessment of ANC coverage and coverage gap. The UDHS-2016 is the seventh round of the Demographic and Health Surveys conducted in the country [27]. The Uganda Bureau of Statistics (UBOS) in collaboration with other agencies implemented the UDHS. The data focus mainly on children under-five years, men and women (15–49 years). A stratified two-stage cluster sampling design was used to collect the data. The first stage is a sample of 696 accessible enumeration areas (i.e., clusters) based on the 2014 population and housing census sample frame. About 77% of the enumeration areas are in rural areas. A fixed number of households (30 per enumeration area) were randomly selected in the second stage. A total of 18,506 women were successfully interviewed. For the multi-country assessment, Demographic and Health Survey (DHS) data (https://dhsprogram.com) from 35 countries in sub-Saharan Africa including Uganda were extracted. The latest available DHS data for each country were extracted in September 2018. A list of these countries, including the data period, is provided under the results section. For all countries, a subset dataset containing women aged 15–49 years were used in this analysis. Of interest in this paper is the most recent ANC service used by women (aged 15–49) with a live birth within the past five years. Other variables of interest, used to assess among other things statistical dominance (for the case of Uganda), include the location of household (urban/rural), education level of the women and household wealth (in quintiles). The DHS data quality checks performed include, among other things, the relevance of each country’s data for inclusion, and the availability and completeness of entries with regards to the key variables of interest. For example, based on this assessment, data from South Africa and Sudan have not been used in this paper. Generally, the DHS data are collected using very similar methodologies across countries, making them comparable. They are widely used and are reliable data for the assessment of maternal and child health statistics in many developing countries.

## Results

The ANC coverage statistics shown in Table 2 indicate that the coverage of only one ANC visit is very low (<3%) in Uganda in 2016. Coverage with at least four ANC visits is slightly greater than 60%. Also, about 28% had only 3 visits while about 2% did not record any ANC visit. Basically, data from 2016 show that about 88% of the women (aged 15–49 years) with a live birth in the past 5 years had attained at least 3 ANC visits based on the most recent live birth experience.

**Table 2:** ANC coverage (most recent live birth) for women aged 15–49 with a live birth within that past five years, Uganda, 2016.

	Proportion (*ρ*_*k*_)	Cumulative proportion (*Y*_*k*_)
4+ ANC visits	0.602* (0.005)	0.602* (0.005)
3 ANC visits	0.275* (0.004)	0.878* (0.003)
2 ANC visits	0.079* (0.003)	0.957* (0.002)
1 ANC visit	0.024* (0.002)	0.981* (0.001)
ANC coverage (*I*_*ANC*_)	0.854* (0.002)	
ANC coverage gap (*G*_*ANC*_)	0.146* (0.002)	

*Notes*: Standard errors in parenthesis

* Estimates are statistically significant at the 1% level

The results from the application of the proposed indices for ANC coverage and coverage gap (i.e., *I*_*ANC*_ and *G*_*ANC*_ respectively) in Uganda (2016) are shown in Table 2. The national average for *I*_*ANC*_ was estimated at 0.854 with a corresponding coverage gap estimated at *G*_*ANC*_ = 0.146. The ANC coverage index *I*_*ANC*_ (and, by implication, *G*_*ANC*_) proposed is additively decomposable by sub-groups. The results of the sub-group decomposition shown in Table 3 indicate that coverage gap is higher in the rural areas (0.154) compared to the urban areas (0.117); for those without any formal education compared to those with some formal education; and for those in poorer wealth quintiles compared to those in richer quintiles.

**Table 3:** Sub–group decomposition of ANC coverage (most recent live birth) for women aged 15–49 with a live birth within that past five years, Uganda, 2016.

	ANC coverage (*I*_*ANC*_)	ANC coverage gap (*G*_*ANC*_)
Urban	0.883* (0.005)	0.117*
Rural	0.846* (0.003)	0.154*
No formal education	0.815* (0.007)	0.185*
Primary education	0.842* (0.003)	0.158*
At least secondary education	0.893* (0.004)	0.107*
Quintile 1 (poorest)	0.822* (0.005)	0.178*
Quintile 2	0.844* (0.005)	0.156*
Quintile 3	0.855* (0.005)	0.145*
Quintile 4	0.863* (0.005)	0.137*
Quintile 5 (richest)	0.887* (0.005)	0.113*
Total	0.854* (0.002)	0.146*

*Notes*: Standard errors in parenthesis

*All estimates are statistically significant at the 1% level

The differences in ANC coverage (Δ*I*_*ANC*_) between groups are shown in Table 4. Except for a few, better-off groups tend to have a significantly higher coverage index compared to worse-off groups (see all positive values in Table 4). However, while some of the values in Table 4 are statistically significant, the statistical test for dominance (Table 5) did not confirm a few of them as statistically significant. For example, $I_{ANC}^{urban}-I_{ANC}^{rural}$ was significantly estimated at 0.037 and this was confirmed by the test of dominance. However, the significant estimates for $I_{ANC}^{Q4}-I_{ANC}^{Q2}$ and $I_{ANC}^{Q2}-I_{ANC}^{Q1}$ in Table 4 are not confirmed in the test of dominance in Table 5.

**Table 4:** Differences in ANC coverage (Δ*I*_*ANC*_) for women aged 15–49 with a live birth within that past five years, Uganda, 2011.

	Rural	Q1 (poorest)	Q2	Q3	Q4	No formal education	Primary education
Urban	0.037* (0.005)						
Q2		0.022* (0.007)					
Q3		0.033* (0.007)	0.011 (0.007)				
Q4		0.041* (0.007)	0.019* (0.007)	0.008 (0.007)			
Q5 (richest)		0.065* (0.007)	0.043* (0.007)	0.032* (0.007)	0.024* (0.007)		
Primary education						0.027* (0.007)	
Secondary+ education						0.078* (0.008)	0.051* (0.005)

*Note*: Figures represent ($I_{ANC}^{row}-I_{ANC}^{column}$) and the standard errors are shown in parenthesis

* Statistically significant at the 1% level

**Table 5:** Dominance analysis for ANC coverage (Δ*Y*_*k*_) for women aged 15–49 with a live birth within that past five years, Uganda, 2011.

	Rural	Q1 (poorest)	Q2	Q3	Q4	No formal education	Primary education
Urban	Dom						
Q2		n-Dom					
Q3		Dom	n-Dom				
Q4		Dom	n-Dom	n-Dom			
Q5 (richest)		Dom	Dom	Dom	Dom		
Primary education						Dom	
Secondary+ education						Dom	Dom

*Note*: Dom = column distribution ($I_{ANC}^{column}$) is dominated by the row distribution ($I_{ANC}^{row}$) at the 5% level of significance

n–Dom = no statistical dominance

## Comparing ANC coverage statistics in sub-Saharan Africa

The proposed ANC coverage index (*I*_*ANC*_) was estimated for African countries with available DHS data. In total, relatively recent DHS data from 35 African counties were used to estimate *I*_*ANC*_. Because the traditional indicator of reporting at least four ANC visits (ANC4+) cannot be directly comparable to the *I*_*ANC*_, country rankings are presented to compare both indictors and to illustrate differences between them.

The results in Table 6 show that as expected, *I*_*ANC*_ > ANC4+ for all countries. Overall, there are some differences between the ranks of countries using ANC4+ and *I*_*ANC*_. Also, as expected, countries like Ghana, Sierra Leone and Swaziland with a relatively high ANC4+ coverage (>80%) are ranked among the better performing countries irrespective of the choice of index. Some countries retained their original ranks. However, in some, the differences in ranks are substantial. Counties like Chad and Ethiopia consistently performed poorly using both ANC4+ and *I*_*ANC*_. Cameroon and West African countries like Nigeria and Benin Republic, for example, were ranked relatively better using the ANC4+ indicator but poorly using *I*_*ANC*_. In Nigeria for instance, while over 50% of the women had attained at least four ANC visits, the proportion that had attained at least 3 ANC visits was less than 60%. On the other hand, an East African country like Rwanda performed better using *I*_*ANC*_ compared to the traditional ANC4+. This was because the proportion of the women with at least 3 ANC visits was about 85%. In comparison with Nigeria, when only the ANC4+ is used, the conclusion will be that Nigeria is performing better than Rwanda but in fact, the proportion of the women with 3 ANC visits is substantially higher in Rwanda than in Nigeria.

**Table 6:** Comparing ANC coverage indicators and associated country ranking.

	ANC coverage^‡^		Country ranking	
Country, year	ANC4+	*I*_*ANC*_	ANC4+ ranking	*I*_*ANC*_ ranking
Ghana, 2016	0.918	0.968	1	1
Sierra Leone, 2013	0.873	0.943	2	2
Swaziland, 2006–07	0.817	0.921	3	3
Gambia, 2013	0.777	0.918	9	4
Liberia, 2013	0.809	0.913	5	5
Namibia, 2013	0.815	0.908	4	6
Gabon, 2012	0.791	0.901	7	7
Sao Tome and Principe, 2008–09	0.786	0.900	8	8
Congo, 2011–12	0.793	0.886	6	9
Lesotho, 2014	0.749	0.883	11	10
Zimbabwe, 2015	0.759	0.875	10	11
Burkina Faso, 2014	0.625	0.869	13	12
Uganda, 2016	0.602	0.854	16	13
Madagascar, 2016	0.588	0.853	17	14
Zambia, 2013–14	0.560	0.850	22	15
Burundi, 2016–17	0.493	0.836	28	16
Malawi, 2015–16	0.508	0.828	27	17
Kenya, 2014	0.578	0.824	18	18
Tanzania, 2015–16	0.509	0.817	26	19
Rwanda, 2014–15	0.439	0.809	31	20
Togo, 2013–14	0.574	0.797	20	21
Senegal, 2016	0.514	0.796	25	22
Comoros, 2012	0.575	0.792	19	23
Benin, 2011–12	0.611	0.780	15	24
Cameroon, 2011	0.630	0.773	12	25
Mozambique, 2015	0.552	0.750	23	26
Guinea, 2012	0.569	0.747	21	27
Congo Democratic Republic, 2013–14	0.483	0.745	29	28
Angola, 2016	0.621	0.739	14	29
Cote d’Ivoire, 2011–12	0.446	0.714	30	30
Niger, 2012	0.329	0.652	33	31
Mali, 2015	0.381	0.629	32	32
Nigeria, 2013	0.528	0.602	24	33
Chad, 2014–15	0.317	0.520	35	34
Ethiopia, 2016	0.319	0.509	34	35

*Source*: Authors’ computation based on Demographic and Health Survey Data from 35 countries

*Notes*: *I*_*ANC*_ is computed using four ANC visits as the recommended minimum number of ANC visits.

^‡^ ANC4+ is the proportion of women aged 15–49 with a live birth within a given period that have attained at least four ANC visits.

In some cases, countries with relatively similar estimates for ANC4+ (E.g. Burkina Faso and Angola with ANC4+ estimated at about 0.62 each) show significant differences in ranking using *I*_*ANC*_. In this case, Burkina Faso was ranked better using *I*_*ANC*_ because it records over 89% of women attaining at least 3 ANC visits compared to 73% reported for Angola.

Within the context of UHC and discussions about progressive realisation, there is a need for accelerating improvements at the margins and especially among disadvantaged population groups [28]. As shown in Table 6, using ANC4+ alone to assess progress in ANC coverage will miss critical improvements that countries make to increase the proportion of women that has attained less than four ANC visits, for example. In general, any country that is able to increase the proportion of women that attain fewer than the recommended minimum number of ANC contacts (e.g. four or eight) [3] are not counted as making any progress unless they increase the number of women that meet that minimum number of contacts. There is an increasing demand for countries to show progressive movements to the ideal of UHC [28] and this is one of the major appeals of the methodology proposed in this paper. In fact, it is the case that women with fewer ANC visits are more likely to come from poorer socio-economic backgrounds [29, 30]. Given the socio-economic inequality in ANC visits, countries can consolidate on any improvement that increases the proportion of women attaining fewer ANC visits to reduce inequality and make substantial contribution to the progressive realisation of maternal health and overall right to health in countries.

While the proposed index (*I*_*ANC*_) used to estimate the results shown in Table 6 presents an improvement over the restrictive ANC4+ index, it does not directly assess the quality or the content including the appropriateness of the timing of the ANC services received. Specifically, in the spirit of the generalised ANC coverage index, $I_{ANC}^{Adj}$, it assumes that quality of care is “perfect” (i.e. *q* = 1) for all ANC contacts. Because this assumption is very unlikely to hold, there is a need to incorporate quality of each ANC contact in estimating ANC coverage. This may depend on the sufficiency of available data in household surveys. Thus, a more robust measure of ANC coverage ($I_{ANC}^{Adj}$), developed in this paper that incorporates a measure of quality will be ideal where data on quality of ANC services are available. If possible, variables that assess quality of service use, across the various dimensions of quality, could be included in routine data collection frameworks to provide for ease of estimating $I_{ANC}^{Adj}$. In fact, the need to assess ANC service quality, or health service quality in general, remains crucial and will be relevant as an area for future research.

The index proposed in this paper focuses on ANC coverage. However, its use may not be restricted to assessing ANC coverage alone. The index could easily be extended to assess progress in other social indicators, for example, education. Here, a minimum number of years of schooling could be determined (e.g. 12 years required to complete secondary education) and used to compute overall coverage and coverage gap indices using all individuals aged at least 18 years.

The study has some strengths and limitations. The ability of the proposed method to incorporate the notion of progressive realisation of maternal health is an advantage. In fact, as noted in the paper, this can be used to assess the progressive realisation of rights to many other phenomena. Also, the basic indices (e.g., ANC coverage gap) are relatively easy to compute and could be communicated to policymakers in ways that are meaningful for policy. Basic quality indicators such as the timing of visits (see Table 1) and content of the services can be incorporated into the proposed index which cannot be done directly using the WHO ‘standard’ or ‘reduced’ ANC coverage indicators that are mainly a counting exercise. While the methodology, especially after adjusting for the quality of care is very appealing, the issue of availability of data on quality of ANC contacts remains a challenge. As a result, the paper has not fully applied the method for quality adjustment directly to empirical data. However, the application in this paper provides preliminary insights into the usefulness of the proposed methodology for assessing ANC coverage globally and it provides the impetus for collecting and analysing quality of ANC services data as part of routine data collection.

## Conclusion

Globally, antenatal care remains an important intervention for improving maternal and child health. While there are debates about the minimum number of ANC visits required for pregnant women to ensure their health and that of their children, the WHO had previously recommended at least four visits (i.e. the ‘reduced’ ANC model) and more recently, the standard ANC model of at least eight ANC contacts, especially in the case of uncomplicated pregnancies. In fact, the WHO’s proposed indicator of attaining at least four ANC visits was used to assess progress towards the MDGs. Although this indicator may be relevant, it does not account for the quality of ANC services received by women. Also, it is not sensitive to any improvements or initiatives that have increased the proportion of pregnant women that attain less than four ANC visits. This paper, using a modified measure of ANC coverage that is additively decomposable by population groups, has shown the importance of accounting for the entire population of pregnant women irrespective of the number of ANC visits. This presents an initial attempt and it allows for an assessment of ANC coverage that tallies with the notion of progressive realisation as entrenched in debates for moving towards UHC. It is envisaged that this paper will, among other things, open the space for rigorous debates about methodologies that are able to demonstrate a country’s progress in (especially by incorporating quality) health service coverage as this represents a very important dimension of UHC.

## Supporting information

S1 Appendix Properties (axioms) of the proposed measure of ANC coverage. (PDF)
