# Supplementary material for: A reassessment of global antenatal care coverage for improving maternal health using sub-Saharan Africa as a case study
Source: PLoS One. 2018 Oct 5;13(10):e0204822. doi: 10.1371/journal.pone.0204822 (PMC6173396; doi:10.1371/journal.pone.0204822)
Supplement: S1 Appendix — (PDF) [file pone.0204822.s001.pdf]

Properties (axioms) of the proposed measure of ANC coverage

John Ele-Ojo Ataguba
Health Economics Unit, School of Public Health and Family Medicine, Health Sciences Faculty, University of Cape Town, Cape Town, South Africa

In the content of poverty, Sen's [1] seminal work provides a set of properties or axioms which a reasonable poverty measure should satisfy. Based on that work and further extensions, it is noted that the proposed ANC coverage (or coverage gap) index satisfies the following properties.

Symmetry or anonymity axiom

Holding every other thing constant, if a new distribution (B) is obtained from an original distribution (A) by a permutation, and the recommended minimum number of ANC visits (m) remains unchanged, then $I_{ANC}^{A}(m) = I_{ANC}^{B}(m)$. This axiom is relevant as it treats all pregnant women equally. Thus, the ANC coverage (or coverage gap) index is estimated based on only information directly related to ANC visits and does not require any other characteristics of the women.

Replication invariance axiom (or population principle)

Holding all else constant, if a new distribution (B) is obtained by replication of the original distribution (A) and the recommended minimum number of ANC visits remains unchanged, then $I_{ANC}^{A} = I_{ANC}^{B}$ and by implication, $G_{ANC}^{A} = G_{ANC}^{B}$.

Focus axiom

Holding all else constant, when a pregnant woman that has attained at least the recommended minimum number of ANC visits (e.g. at least four ANC visits) reports more ANC visits, I_{ANC} (and G_{ANC}) will remain unchanged. This property means that any further ANC visits among those that have attained the recommended minimum number of ANC visits (m) would have no effect on the measure of ANC coverage or coverage gap. It is only any increase or decrease in the number of ANC visits

among those that have not attained the minimum number of visits that has an impact on I_{ANC} and G_{ANC}.

Scale invariance axiom

Holding all else constant, if all ANC visits (including the recommended minimum number of ANC visits) are multiplied by the same proportion $\delta > 0$, then I_{ANC} will remain unchanged.

Monotonicity axiom

Holding all else constant, when a pregnant woman with less than the recommended minimum number of ANC visits (e.g. four ANC visits) attains at least one more ANC visit, the ANC coverage index (I_{ANC}) improves while the ANC coverage gap (G_{ANC}) reduces.

Transfer axiom

Because it is not literally possible to transfer ANC visits from one woman to another, a modification of the axiom is discussed here. This is also consistent with discussions regarding non-monetary dimensions of poverty. This means that increasing the number of women with fewer ANC visits will increase I_{ANC} (by monotonicity) but the increment will be less than that which will result from increasing the number of pregnant women with higher ANC visits. These ANC visits should all be less than the recommended minimum number of visits (m). This axiom implies that the impact of fewer visits on I_{ANC} is less than the impact of more visits if we are focusing on women with less than the recommended minimum number of ANC visits.

Normalisation axiom

This is a trivial axiom which simply means that $I_{ANC} = 1$ ($G_{ANC} = 0$) if all women aged 15-49 with a live birth within a given period had attained the recommended minimum number of ANC visits. In the

case of four ANC visits (as a bare minimum), this means that 100% of the women had attained at least four ANC visits within the reference period.

Sub-group decomposition (additive)

Holding all else constant, overall ANC coverage index (I_{ANC}) can be obtained as the weighted average of ANC coverage of all mutually exclusive groups, where the weights are the relative shares of the groups in the total population. E.g., if two mutually exclusive groups (A and B) are formed with their respective ANC coverage given as I_{ANC}^{A} and I_{ANC}^{B} and sub-populations n_A and n_B where $n_A + n_B = N$, then I_{ANC} is additively decomposable as $I_{ANC} = \frac{1}{N}\left(n_A\left(I_{ANC}^{A}\right) + n_B\left(I_{ANC}^{B}\right)\right)$. Generally, this can be extended to any arbitrary number of groups ($g \geq 2$) such that $I_{ANC} = \frac{1}{N}\sum_g n_g\left(I_{ANC}^{g}\right)$.

Reference

1. Sen A. Poverty: an ordinal approach to measurement. Econometrica. 1976;44(2):219-31
